# Supplementary material for: Causal Relationship between Aging and Anorexia Nervosa: A White-Matter-Microstructure-Mediated Mendelian Randomization Analysis
Source: Biomedicines. 2024 Aug 16;12(8):1874. doi: 10.3390/biomedicines12081874 (PMC11351342; doi:10.3390/biomedicines12081874)
Supplement: Supplementary file 1 [file biomedicines-12-01874-s001.zip › Supplementary Figure/Figure S4/Singe SNP plot of MR analysis for TL to ubm-b-1591.pdf]

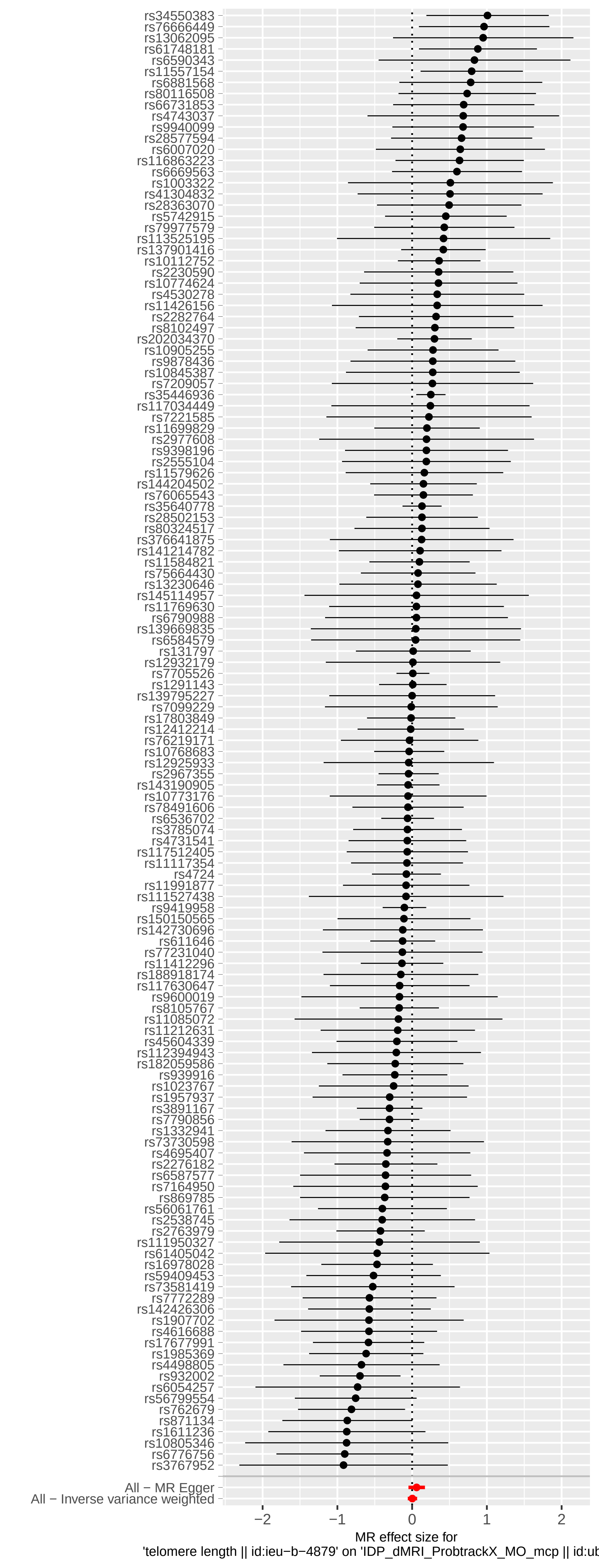
rs34550383
rs76666449
rs13062095
rs61748181
rs6590343
rs11557154
rs6881568
rs80116508
rs66731853
rs4743037
rs9940099
rs28577594
rs6007020
rs116863223
rs6669563
rs1003322
rs41304832
rs28363070
rs5742915
rs79977579
rs113525195
rs137901416
rs10112752
rs2230590
rs10774624
rs4530278
rs11426156
rs2282764
rs8102497
rs202034370
rs10905255
rs9878436
rs10845387
rs7209057
rs35446936
rs117034449
rs7221585
rs11699829
rs2977608
rs9398196
rs2555104
rs11579626
rs144204502
rs76065543
rs35640778
rs28502153
rs80324517
rs376641875
rs141214782
rs11584821
rs75664430
rs13230646
rs145114957
rs11769630
rs6790988
rs139669835
rs6584579
rs131797
rs12932179
rs7705526
rs1291143
rs139795227
rs7099229
rs17803849
rs12412214
rs76219171
rs10768683
rs12925933
rs2967355
rs143190905
rs10773176
rs78491606
rs6536702
rs3785074
rs4731541
rs117512405
rs11117354
rs4724
rs11991877
rs111527438
rs9419958
rs150150565
rs142730696
rs611646
rs77231040
rs11412296
rs188918174
rs117630647
rs9600019
rs8105767
rs11085072
rs11212631
rs45604339
rs112394943
rs182059586
rs939916
rs1023767
rs1957937
rs3891167
rs7790856
rs1332941
rs73730598
rs4695407
rs2276182
rs6587577
rs7164950
rs869785
rs56061761
rs2538745
rs2763979
rs111950327
rs61405042
rs16978028
rs59409453
rs73581419
rs7772289
rs142426306
rs1907702
rs4616688
rs17677991
rs1985369
rs4498805
rs932002
rs6054257
rs56799554
rs762679
rs871134
rs1611236
rs10805346
rs6776756
rs3767952
All − MR Egger
All − Inverse variance weighted
−2
−1
0
1
2
MR effect size for
'telomere length || id:ieu−b−4879' on 'IDP_dMRI_ProbtrackX_MO_mcp || id:ub
